# What Affects the Economic Resilience of China’s Yellow River Basin Amid Economic Crisis—From the Perspective of Spatial Heterogeneity

**DOI:** 10.3390/ijerph19159024

**Published:** 2022-07-25

**Authors:** Liangang Li, Pingyu Zhang, Chengxin Wang

**Affiliations:** 1 College of Geography and Environment, Shandong Normal University, Jinan 250358, China; llg911208@163.com; 2 Northeast Institute of Geography and Agroecology, Chinese Academy of Sciences, Changchun 130002, China; zhangpy@iga.ac.cn; 3 College of Resources and Environment, University of Chinese Academy of Sciences, Beijing 100049, China

**Keywords:** regional economic resilience, spatial heterogeneity, spatiotemporal evolution, influence mechanism, Yellow River Basin, China

## Abstract

This paper contributes to the study of regional economic resilience by analyzing the dynamic characteristics and influence mechanisms of resilience from the perspective of spatial heterogeneity. This paper focuses on the resistance and recoverability dimensions of resilience and analyzed the dynamic changes in economic resilience in China’s Yellow River Basin in response to the 2008 economic crisis. The multi-scale geographical weighted regression model was utilized to examine the effect of key factors on regional economic resilience. Our findings show the following: (1) The resistance of the Yellow River Basin to the financial crisis was high; however, the recoverability decreased significantly over time. (2) The spatial heterogeneity of driving factors was significant, and they had different effect scales on economic resilience. Related variety, government agency, environment, and opening to the global economy had a significant effect on economic resilience only in a specific small range. Specialization, unrelated variety, and location had opposite effects in different regions of the Yellow River Basin. (3) Specialization limited the area’s resistance to shock but enhanced the recoverability. Related variety improved regional economic resilience. Unrelated variety was not conducive to regional resistance to shock and had opposite effects on the recoverability in different regions. (4) Government agency and financial market promoted regional economic resilience. Environment pollution and resource-based economic structure limited regional economic resilience. Opening to the global economy and urban hierarchy limited regional resistance to shock, but strong economic development had the opposite effect of improved regional resistance. The location in the east of the Yellow River Basin enhanced the recoverability; however, the location in the west limited the recoverability.

## 1. Introduction

As complex dynamic systems, regional economic systems are frequently affected by uncertain shocks such as international financial crisis and COVID-19 [1,2,3]. How to deal with uncertain shocks, resist the impact of the shocks, and quickly restore development has become the key to maintaining the healthy and sustainable development of cities in the current highly uncertain shock environment [4,5,6]. However, regional (urban) economic systems show significant spatial differences in coping with shocks. Specifically, some regions have been seriously affected by shocks, resulting in stagnation of or a decline in economic development. Interestingly, some cities can resist the negative impact of shocks and achieve rapid economic recovery in a short time through adjustment [7,8,9]. The different performances of urban response to shocks have led to extensive discussions in economic geography and regional science on how to cope with shocks and to what extent regional economic systems can recover or even transform [10,11,12,13]. Regional economic resilience is considered to be the key to explaining such differences [14,15]. Especially under the COVID-19 epidemic shock, regional economic resilience has become a buzzword in the current academic and policy circles both in developed and emerging economies [3,16,17,18]. Regional economic resilience addresses the regional ability to resist and recover from external shocks and, broadly speaking, to adapt to uncertain contexts for maintaining the evolving abilities for economic development and growth [4,6,10]. Regional economic resilience provides a new perspective for analyzing the evolution mechanism of regional systems under uncertain shock environments [3].

The Yellow River Basin is an important ecological region and major economic zone in China. It spans eight provinces and has significant differences in different scale units within the region. The Yellow River Basin includes the eastern coastal areas and the central and western regions of China. Regional economic development has long depended on resource-based industries and opening to the outside world [1,19], and investment has a great pulling effect on regional development. This has played an important role in the economic development of the Yellow River Basin, but it has also caused serious environmental pollution. However, in the context of the financial crisis, this over-reliance on specific investment and poor environments may limit the regional economic resilience to the shock [20]. This is mainly because, under the influence of the financial crisis, foreign trade declined, foreign capital began to withdraw capital to reduce losses, and the desire for domestic capital investment declined. After the shock, China entered a high-quality development stage, and the environmental situation has since become an important force to support economic development. The reduction in investment and the weak environment limit the ability of the Yellow River Basin to cope with the shock, and the economic development is slow.

The weak ecological environment and extensive development model of the Yellow River Basin lead to its extreme vulnerability to external shocks such as the financial crisis [21], resulting in the interruption of the regional development path and falling into the recession track. The Yellow River Basin is a typical vulnerable area to the financial crisis. Under the background of China’s national strategy of ecological protection and high-quality development of the Yellow River Basin in 2019, how to deal with uncertain shocks and maintain stable economic development in the Yellow River Basin has become the key to high-quality development. There is an urgent need to study the characteristics of economic resilience in the Yellow River Basin and explore the differences in the influence mechanism of regional economic resilience from the perspective of basin heterogeneity. This paper attempts to explore ways to improve the ability of the Yellow River Basin to deal with external shocks so as to maintain the sustainable economic development of the Yellow River Basin in the event of uncertain shocks in the future.

Therefore, in this paper, we attempt to contribute to the study of regional economic resilience in terms of dynamic measurement of regional economic resilience and the spatial heterogeneity of the influence mechanism. Based on the dynamic perspective, we took the prefecture-level cities in the Yellow River Basin of China as the research object. We conducted a dynamic analysis on the level of economic resilience of the Yellow River Basin in the face of the 2008 international financial crisis. We tried to explore the spatiotemporal evolution law of regional economic resilience. On this basis, we comprehensively considered structural factors, agency-based factors, resource-based economic structure, environment, and urban hierarchy factors and provide empirical analysis on the spatial differences in influencing factors of regional economic resilience under different spatial scales. This paper attempted to explore the influence mechanism of regional economic resilience so as to provide a theoretical reference for the sustainable and stable development of the Yellow River Basin under uncertain shocks.

The remainder of this paper is organized as follows. Section 2 reviews the relevant literature. Section 3 introduces the study area, methodology, and influence factors of regional economic resilience. After showing the spatiotemporal evolution characteristics of regional economic resilience in Section 4, Section 5 discusses the determinants of regional economic resilience in the Yellow River Basin. Section 6 concludes the paper.

## 2. Review of Literature

At present, the world is facing various uncertainties. Such uncertain events may cause serious consequences and eventually lead to the emergence of shocks or risks such as a financial crisis [22,23]. The shocks or risks will have an important impact on the investment decisions of different actors. The shocks may lead to expected investment failure and limit regional sustainable development [20]. Regional economic resilience is considered to be the key factor to explain a region’s response to uncertain shocks or risks. How to reduce the possibility of occurrence of uncertain risks, mitigate the negative impact of risks, reduce the vulnerability of the economic system, and improve the regional economic resilience to cope with future shocks is the key to maintaining regional sustainable and stable development under the current highly uncertain environment [20,23,24].

Resilience was first used to represent the rebound of physical systems after experiencing shocks. Holling introduced the concept of resilience into ecology, indicating the ability of ecosystems to recover to a single equilibrium state after a shock [25]. Subsequently, the connotation of resilience was extended to multiple equilibrium states, indicating the ability of a region to return to the original stable state or enter a new stable state after a shock [26,27]. From the perspective of evolution, the concept of evolutionary resilience was put forward [6,10,28]. Evolutionary resilience emphasizes that the system is always in the never-ending process of nonlinear dynamic change, and resilience reflects the long-term adaptation process of the system in the uncertain shock environment [11,29]. Regional economic resilience refers to the ability of a regional economic system to resist shocks in the face of external shocks, to mitigate the impact of shocks, and to recover the pre-shock development trajectory of the region or shift to a new and better development trajectory through adaptation [3,26].

The concept of regional economic resilience involves the discrimination of adaptation and adaptability. Adaptation starts from the path dependence and emphasizes the structure and mode formed by the regional economic system in its long-term historical development process. This historical shaping has strong adaptation. In a short time, when a region is subject to external shocks, the path dependence of the regional system can maintain the original development path of the region or the path renewal based on the original foundation and emphasize the resistance, recovery, and renewal of the region from the shock [11,17]. Additionally, adaptability is more about path breakthrough, emphasizing that when the regional economic system is subject to external shocks, under the agency of the state and other governments, it can break the original development path, establish a new development path, achieve path breakthrough, and maintain the new development of the region in the long term, thus emphasizing the re-orientation [10,30]. Therefore, the explanation of regional economic resilience in our paper is more from the perspective of adaptation in a short period of time, emphasizing the resistance and recovery of the region to the shock.

Resilience measurement is mainly based on two methods: constructing a single index or an indicator system. A resilience index is mainly aimed at “sudden shocks” (especially the 2008 international financial crisis). It is measured through core variables such as GDP and employment [31,32,33]. However, most of the existing studies chose provincial units for research, and this ignores the urban differences within province [34,35]. Most of the studies were analyzed from the static perspective [29,36]. Resilience is measured by the variables at the beginning and end of the shock, which only reflects the final resilience in this period. However, the economic resilience at different times in this period is not measured, and the change process of economic resilience in this period cannot be reflected. There are few studies from the dynamic perspective, which cannot fully reflect the influence process of external shocks on regional economy [37]. The index system method is mainly aimed at the measurement of regional economic resilience under “slow-burn” shocks [38,39], involving crisis contexts such as resource depletion and population contraction. However, there is no consensus on indicators, resulting in large differences in index systems [40].

The influencing factors of regional economic resilience are the focus of empirical research. Influenced by the evolutionary economic geography, the influencing factors of regional economic resilience mainly focus on structural factors such as industrial structure and industrial development models [10,12,37]. They also focus on the impact of regional basic industry composition, diversification, and specialization on regional economic resilience. It is generally believed that regions with more significant manufacturing industries are seriously affected by financial crisis [29]. Specialized structures will accelerate shock diffusion. Diversified structures can play the role of “shock absorber” and reduce the influence [3,6,8,37]. Diversified structures also involve the discussion of related diversification and unrelated diversification [17]. In addition, the role of agency-based factors such as entrepreneurship and government support has been paid an increasing amount of attention. It is found that agency-based factors can affect economic resilience through adjusting the structural factors [3,4,12,41], but the role of government agency is often ignored in the existing research [3,17].

There are few studies on economic resilience at the watershed scale [1]. Watershed units cover a wide range, and the existing studies did not pay attention to the spatial heterogeneity of watershed-scale objects. Goodchild believes that scale is the most important topic in geographic information science [42]. The influence mechanism of a certain socio-economic phenomenon is often determined by multiple spatial processes of different scales. However, the existing studies ignored the spatial heterogeneity of influencing factors and often assumed that all influencing factors play a role in the whole study area [34,37]. It should be noted that some influencing factors may only have a significant impact in a small area but not in other areas. Some influencing factors have positive effects in a certain area but negative effects in another specific area. This is also the focus of our paper.

Based on the framework of regional economic resilience proposed by Martin [8,13,26] combined with the characteristics of our study area and considering spatial heterogeneity, we put forward the theoretical analysis framework of this paper. Regional economic resilience is the ability of a region to adapt to external uncertain shocks such as a financial crisis. It is mainly reflected in two dimensions: resistance and recoverability. Resistance reflects the vulnerability of a region to a financial crisis and the depth of impact. High resistance indicates that the region is less affected by the shock. Recoverability reflects the regional recovery after the financial crisis. High recoverability indicates that the region can actively adapt to the environmental changes after the shock and achieve recovery development through path adjustment and transformation.

## 3. Study Area and Possible Influencing Factors

### 3.1. Study Area and Period Division

**Figure 1:**
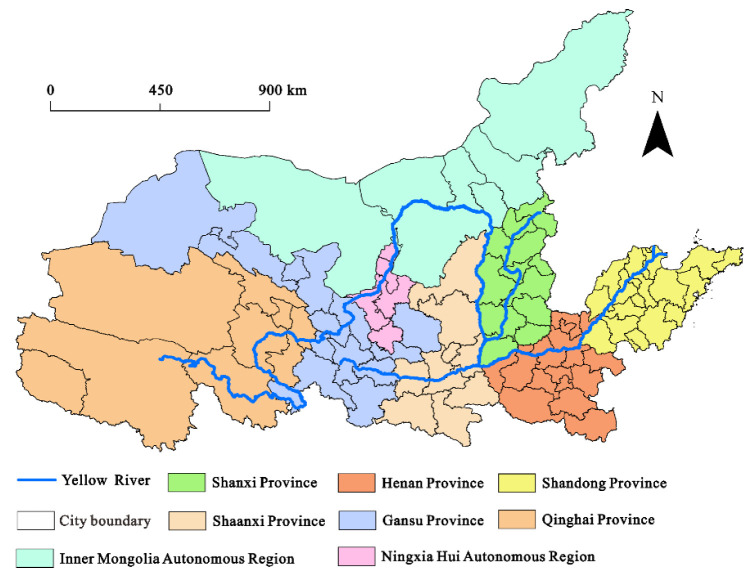
The location of the Yellow River Basin in China.

The Yellow River Basin in China mainly covers eight provinces and regions (Figure 1), including 91 prefecture-level cities [43]. In 2018, the population of the Yellow River Basin accounted for 23.8% of China, while the GDP accounted for only 20.35% of China, and the economic development was slow. Moreover, there are many resource-based cities in the Yellow River Basin, accounting for 46%.

**Figure 2:**
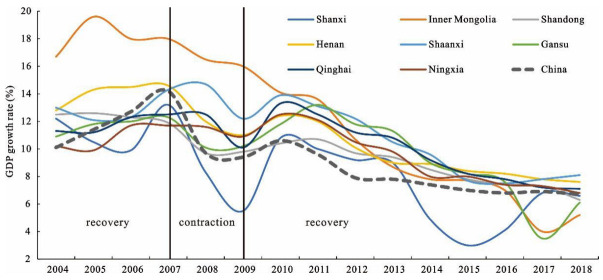
The GDP growth rate in 2004–2018.

Considering data availability and administrative consistency, we used the GDP growth rate to divide the economic development stages of the Yellow River Basin before and after the 2008 financial crisis, providing a basis for the measurement of regional economic resilience [12,29]. The GDP growth rate of China and the eight provinces is shown in Figure 2.

It can be found that, before the 2008 financial crisis (2004–2007), the GDP rate showed a continuous upward trend and reached its peak in 2007, which was the recovery period of the region facing the last shock. However, after the 2008 international financial crisis, the GDP rate declined rapidly, reaching the first trough in 2009, and the international financial crisis basically ended after 2010. Therefore, the period 2008–2009 was the contraction period of the Yellow River Basin facing the financial crisis. The “4 trillion” support policy proposed by China in response to the financial crisis played an important role in the economic recovery development, making the GDP rate rise briefly in 2010. However, China then ended the high-speed growth trend, paid attention to the high-quality development of the economy, and stepped into a medium–high-speed growth trend. After 2010, the GDP rate showed a downward trend. This downward trend was mainly due to the influence of environmental change caused by the 2008 financial crisis and transformation of China’s economic development mode, which still belongs to the recovery period after the financial crisis. Therefore, we believe that 2010–2018 is the recovery period of the Yellow River Basin after the financial crisis.

### 3.2. Research Methods

#### 3.2.1. Measurement of Regional Economic Resilience

In this paper, the regional economic resilience is measured by constructing the resistance and recoverability index [8,12], which is calculated by comparing the change of urban GDP with the expected change.

The calculation of the expected change of urban economic output in the contraction (2007–2009) or recovery (2009–2018) period is as follows:

(1) $${\left(\Delta R_{i}{}^{t+k}\right)}^{expected}=\sum_{j}^{n}R_{ij}{}^{t}\times G_{n}{}^{t+k}$$

where ${\left(\Delta R_{i}{}^{t+k}\right)}^{expected}$ represents the expected change of economic output of city *i* in the contraction or recovery period (*t + k*), $R_{ij}{}^{t}$ represents the economic output of industry *j* of city *i* at starting time *t*, and $G_{n}{}^{t+k}$ represents the change rate of national economic output in *t + k* time.

The calculation of resistance and recoverability is as follows:

(2) $$Resistance=\frac{\left(\Delta R_{i}{}^{contraction}\right)-{\left(\Delta R_{i}{}^{contraction}\right)}^{expected}}{\left|{\left(\Delta R_{i}{}^{contraction}\right)}^{expected}\right|}$$

where $\left(\Delta R_{i}{}^{contraction}\right)$ represents the actual change of economic output of city *i* during the contraction period, and ${\left(\Delta R_{i}{}^{contraction}\right)}^{expected}$ represents the expected change of economic output of city *i* during the contraction period.

(3) $$Recoverability=\frac{\left(\Delta R_{i}{}^{restore}\right)-{\left(\Delta R_{i}{}^{restore}\right)}^{expected}}{\left|{\left(\Delta R_{i}{}^{restore}\right)}^{expected}\right|}$$

where $\left(\Delta R_{i}{}^{restore}\right)$ represents the actual change of economic output of city *i* during the recovery period, and ${\left(\Delta R_{i}{}^{restore}\right)}^{expected}$ represents the expected change of economic output of city *i* during the recovery period.

Resistance (recoverability) greater than 0 means that the resistance (recoverability) of the city in the face of shock is higher than the national average.

#### 3.2.2. Multi-Scale Geographical Weighted Regression (MGWR)

MGWR considers the problem of spatial heterogeneity and improves the geographic weighted regression by allowing different bandwidths of each variable [44]. At the same time, it provides the influence scale of different variables, which can analyze the effect of each variable on the dependent variable at different scales and obtain more reliable estimation results. The MGWR model can reflect whether an independent variable has a significant impact on the dependent variable in the whole study area, whether an independent variable has a significant impact on the dependent variable in a specific range within the study area, and whether an independent variable has opposite or different impacts on the dependent variable in different ranges within the study area. The calculation of the MGWR model is as follows:

(4) $$y_{i}=\sum_{j=1}^{k}\beta_{bwj}\left(u_{i},v_{i}\right)x_{ij}+\varepsilon_{i}$$

where *β_bwj_* represents the regression coefficient of the *j*-th variable, *b_wj_* represents the bandwidth used by the regression coefficient of the *j*-th variable, (*u_i_*, *v_i_*) represents the spatial coordinates of region *i*, *x_ij_* represents the observed value of the *j*-th variable of region *i*, and *ε_i_* is the random perturbation term.

### 3.3. Possible Influencing Factors of the Yellow River Basin’s Economic Resilience

Regional economic resilience is affected by many factors. The industrial structure is considered to be a key factor affecting regional economic resilience and has been widely discussed [27,45]. However, the role of government agency is often ignored [3,6]. The proportion of state-owned enterprises is significant, and the regional economic development is seriously affected by the government’s macro-control. Therefore, the economic resilience of the Yellow River Basin to cope with uncertain shocks may be more deeply affected by government agency. However, the influence mechanism of regional economic resilience is not clear. It is urgent to further explore the role of different structural and agency-based factors in regional economic resilience [12]. According to the literature, we summarize the main factors affecting regional economic resilience as follows.

#### 3.3.1. Industrial Structure

The industrial structure is a key factor affecting regional economic resilience, and a change in the industrial structure will affect the resistance and recoverability of a regional economy to shocks [26,46]. The specialized structure is more likely to be exposed to shocks, which is prone to the shock diffusion of “pulling one hair and moving the whole body” [17]. The vulnerability of regional economic systems is high, meaning that rapid fluctuations in the regional economy can be easily caused. However, in the recovery stage, the specialized industrial structure may be more conducive to regional recovery, mainly because the specialized structure can help to improve the implementation effect of specific policies and guide the rapid transformation of specific industries to achieve recovery and development.

A diversified structure plays the role of “shock absorber” [3,17,47]. When the shock has a specific industrial orientation, other types of industries are less affected and can complement and maintain economic development [12,29]. However, the effect of diversified structures on regional economic resilience is not clear, which mainly involves the discussion of related variety and unrelated variety. Related variety focuses on the technology substitution or complementarity between different industries, and different industries have similar knowledge or capability bases [6,8]. Related variety takes into account industrial cooperation while dispersing the shock, which is considered to be the core of developing long-term economic resilience [11,26]. Unrelated variety can block the spread of specific shocks due to the lack of industrial linkages. In the short term, it may be conducive to the dispersion of shocks, but in the long term, it is not conducive to industrial innovation and economic resilience [29].

#### 3.3.2. Opening to the Global Economy

The degree of openness to the global economy includes the degree of cooperation outside the region and can also represent the degree of embedding in the global economy or participating in the global division of labor. A high degree of openness to the global economy means that the region can attract external funds and technologies, export products and services to the outside world, and improve the efficiency of the regional economic system. After the shock, it can adapt to the new environment in time and improve the regional ability to deal with the shock [34,48,49]. However, opening to the global economy plays a double-edged sword role in regional economic resilience. When the shock seriously affects the regional import and export departments, the regions with a high degree of openness to the global economy may be more seriously impacted, which limits the regional ability to deal with the shock [4,31].

#### 3.3.3. Government Agency

As a key factor affecting regional economic resilience, government agency can even determine regional economic resilience [3,17,50]. Government agency, such as regulation and control, can guide the optimization and adjustment of the regional industrial structure to change the development mode and attract high-quality talents and then affect the level of regional economic resilience to cope with the shock [4,40,51]. After the shock, the government support or investment play can limit the negative influence of the shock to the greatest extent. The government can provide support for the recovery and development of enterprises or industries [26]. Government agency can provide policy support for the regional economic system to update the development path or create a new path and improve the regional economic resilience to the shock [3,52].

#### 3.3.4. Financial Market

The financial market of the regional economic system is an important variable to deal with the financial crisis. It determines the development of the regional economy under uncertain shocks through the process of investment or resource utilization [20]. Regions with a good financial market may have a better ability to cope with shocks. This is mainly because a good financial market helps to attract capital. After the shock, it can provide enough capital support for enterprises to cope with the shock, help the industry through the difficult period, and then enhance the regional resilience. At the same time, a good financial market can provide stable capital investment for industrial adjustment, improve or transform resource utilization efficiency, promote regional innovation output and the pace of industrial transformation, and then enhance regional resilience [26,34,48].

#### 3.3.5. Resource-Based Economy

Resource-based cities in the Yellow River Basin account for 46%. Studies have shown that compared with other cities, resource-based cities have high vulnerability and poor adaptability to shocks [3,11,12]. The development of cities in the Yellow River Basin mostly depends on resource-based industries, which are greatly affected by market prices. Under the 2008 international financial crisis, the price of raw materials seriously declined. The development path of the Yellow River Basin relying on resource-based industries may limit the ability of the region to resist shocks. With China entering a high-quality development stage and the intensification of resource depletion, the development of the resource-based economy in the Yellow River Basin has been severely restricted. Strong path dependence has hindered the industrial breakthrough and transformation, which may limit the regional recovery and transformation. In a word, the resource-based economy in the Yellow River Basin may limit the resilience [3,12,37].

#### 3.3.6. The Environmental Condition of the Yellow River Basin

The ecological environment of the Yellow River Basin is relatively fragile, facing serious resource and environmental problems. As China enters the stage of high-quality development, the environment has become the key to restricting the sustainable development of regional economy [1,19,53]. The development mode of the Yellow River Basin over-relying on resource-based industries has aggravated environmental pollution and restricted the entry of high-end talents and high-tech industries. There are many industries with high energy consumption and high pollution, which are more vulnerable to fluctuations in the international market, which may lead to the low ability of the region to cope with the financial crisis. With the transformation of the regional development mode, the regions with improved environmental conditions after the shock have realized industrial adjustment and transformation, attracting talents, new technologies, and new industries. The regions can adapt to the new environment and achieve rapid recovery and development.

#### 3.3.7. Urban Hierarchy

The Yellow River Basin covers a large area, and the urban hierarchy varies greatly. The hierarchy difference between cities may be a factor affecting the resilience. In the context of economic globalization, cities with a high degree participate more in the regional division of labor and have close external relations. The negative impact of the international financial crisis may have a more serious influence on the economy of high-grade cities through the process of economic globalization. However, after the shock, high-grade cities may attract talents and capital through their attraction, actively participate in the regional division of labor in the post-financial crisis era, and quickly restore regional economic development.

In addition, regional economic resilience is also related to the level of economic development. It is generally believed that areas with good economic development can provide funds, infrastructure, and technical support for the region to cope with shocks [4]. Areas with a high urbanization level can attract the agglomeration of resource elements, and their modern industrial structure and development mode may help the region adapt to the transformation in time after the shock [54].

Based on the existing literature, we selected industrial specialization, industrial diversification, degree of openness, government agency, environment, resource-based economy, and urban hierarchy as key elements and took economic development level and modernization level as control variables to explore the influence mechanism of economic resilience in the Yellow River Basin. The connotation and calculation method of each variable are shown in Table 1.

Specialization (SPC) and diversification (RV, U-RV) are calculated by the number of employed persons in urban units by industry in various regions. The calculation of specialization index is:

(5) $$SPC_{i}=\sum_{j=1}^{k}\left|V_{i,j}-V_{j}\right|$$

where *V_i__,j_* represents the proportion of the employment of industry *j* in region *i* in the total employment of the region. *V_j_* represents the proportion of the employment of industry *j* in the total employment of the Yellow River Basin.

Economic diversification is represented by related variety (RV) and unrelated variety (U-RV) respectively. The calculation of related variety is as follows:

(6) RVi,t=∑j=1kPi,j×Ei,j;Ei,j=∑s=1n(Pi,sPi,j)×ln(Pi,jPi,s)

where *P_i__,s_* is the proportion of employment in the *s*-th industry of city *i* at time *t*, and *n* is the number of all industries. In the paper, *n* is 19, *P_i__,j_* is the proportion of employment in the *j*-th major department of city *i* at time *t*, and *k* is the number of major departments, which is divided into six major departments on the basis of 19 industries in the paper.

The calculation of unrelated variety is:

(7) U−RVi,t=∑j=1kPi,j×ln(1Pi,j)

Considering the serious lack of data in some cities, only 87 cities were selected for analysis in the influencing factor analysis part.

### 3.4. Data Sources

Our data were derived from the China City Statistical Yearbook for 2004–2018 and the relevant province’s statistical yearbook.

## 4. Spatiotemporal Evolution Characteristics of Regional Economic Resilience

### 4.1. Temporal Evolution Characteristics

**Figure 3:**
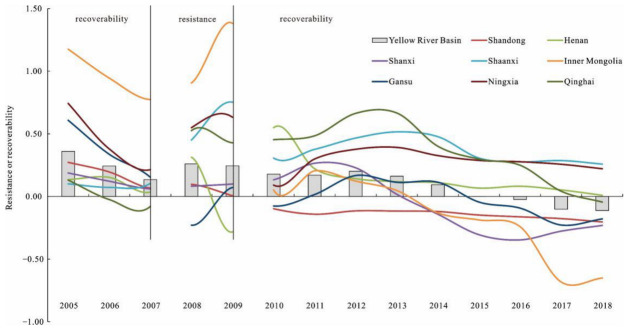
Average economic resilience of the Yellow River Basin.

Based on Equations (2) and (3), we dynamically measured the resistance and recoverability levels of the Yellow River Basin in the face of the 2008 international financial crisis. At the same time, in order to compare the economic resilience of the Yellow River Basin to this shock, we also measured the recoverability of the region to the previous shock before 2008. In order to further reflect the temporal evolution of the overall economic resilience of the Yellow River Basin, we calculated the average value of the resistance and recoverability levels of the Yellow River Basin and eight provinces from 2005 to 2018. The results are shown in Figure 3.

The results show that before the 2008 financial crisis, that is, during the period of 2005–2007, the average recoverability of the Yellow River Basin and most provinces was greater than 0, indicating that the economy of the Yellow River Basin had recovered. In the stage of resistance to the 2008 financial crisis (2008–2009), the average resistance of the Yellow River Basin and most provinces was greater than 0, indicating that the Yellow River Basin was less affected by the shock than the national average level, and the regional economy had high resistance to the shock except for Henan. This is mainly because of the “4 trillion” support policy put forward by the state in response to the international financial crisis, which buffered the impact of the shock on the Yellow River Basin during this period.

During the recovery phase (2010–2018), the recoverability of the Yellow River Basin and all provinces showed a downward trend, and in recent years, the average recoverability of the Yellow River Basin has been less than 0. From 2010 to 2018, the recoverability level of the Yellow River Basin decreased by 163%. This shows that in the recovery stage, under the background of “three periods superimposed” and China’s entry into the high-quality development stage compared with the national average level, the recoverability of the Yellow River Basin was weak, the ability of the regional economy to adapt to the new environment was weak, and the regional transformation and development were slow. Except for Shaanxi, Ningxia, and Henan, the average recoverability of the other provinces was less than 0 in 2018. For a long period of time, the Yellow River Basin could not adapt to the new environment during the recovery period, making it difficult to update or break through the path of regional economic development, and the speed of regional economic development was lower than the national average.

There were significant differences in economic resilience among different provinces. This spatial difference in economic resilience may lead to spatial differences in the mechanism of influencing factors.

### 4.2. Spatial Evolution Characteristics

**Figure 4:**
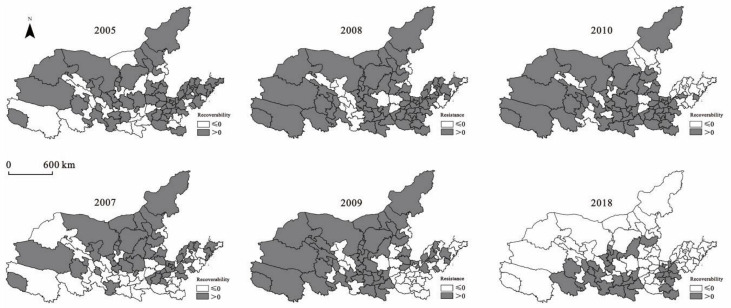
The economic resilience of the Yellow River Basin.

The level of resistance or recoverability of the Yellow River Basin in 2005, 2007, 2008, 2009, 2010, and 2018 was selected for visual expression to reflect the spatial evolution characteristics of economic resilience in the Yellow River Basin. The specific results are shown in Figure 4.

Figure 4 shows that the recoverability of the Yellow River Basin was high from 2005 to 2007 but showed a downward trend. The proportion of cities with recoverability less than 0 rose from 31% in 2004 to 47% in 2007. From 2008 to 2009, the resistance of the Yellow River Basin to the financial crisis was higher than the national average, with average resistances of 0.26 and 0.24, respectively, but the resistance level in the eastern part of the Yellow River Basin decreased significantly. Cities with high resistances showed significant characteristics of spatial agglomeration. From 2008 to 2009, the proportion of cities with resistance less than 0 rose from 22% to 44%. From 2010 to 2018, the recoverability of the Yellow River Basin showed a significant downward trend. The recovery and development of the Yellow River Basin were slow after the shock. The average recoverability decreased from 0.18 to −0.11, decreasing by 162.9%. From 2010 to 2018, the proportion of cities with recoverability less than 0 rose from 33% to 65%. Cities with high recoverability were mainly concentrated in the south of the Yellow River, while other regions had low recoverability over time. The regional development path after the shock could not adapt to the new environment. At the same time, it was found that the resistance or resilience of the Yellow River Basin showed characteristics of spatial differences.

## 5. Determinants of Economic Resilience in the Yellow River Basin

### 5.1. Model Comparison and Scale Analysis

Before the regression of the model, we carried out variance inflation factor (VIF) test on the selected influencing factors. The results show that the VIF values of all variables were less than 4, indicating that there was no multicollinearity among the influencing factors, so regression analysis could be carried out. Table 2 shows the regression results of the OLS model and MGWR model in the 2008–2009 resistance period and 2010–2018 recovery period, respectively. The results show that the goodness-of-fit R^2^ and log-likelihood (log-L) values of the MGWR model were higher than those of OLS model, and the Akaike information criterion (AIC) value was lower than that of OLS, indicating that the regression result of the model was more accurate after considering spatial heterogeneity. At the same time, it was found that the heterogeneity scale plays an extremely important role in the influence mechanism of regional economic resilience. Some variables only had an impact on economic resilience within a specific range. Once the specific range was exceeded, the impact of the variable disappeared. In other words, the action process of different factors on regional economic resilience runs on different spatial scales.

The MGWR results show that in the resistance stage (2008–2009), the bandwidth of specialization, related variety, opening to the outside world, government agency, and GDP were small. They had high spatial heterogeneity. Beyond this scale, the regression coefficient changed dramatically. In the recovery stage (2010–2018), the bandwidth of constant and unrelated variety were small. The constant represents the impact of different locations on regional economic resilience when other independent variables are determined. Its bandwidth was 44, indicating that the recoverability of the Yellow River Basin was the most sensitive to location and had a great impact in a local range. The impact of influencing factors on economic resilience can be shown by the coefficient spatial distribution map below.

### 5.2. Spatial Pattern Analysis of Influencing Factors

#### 5.2.1. Resistance in 2008–2009

**Figure 5:**
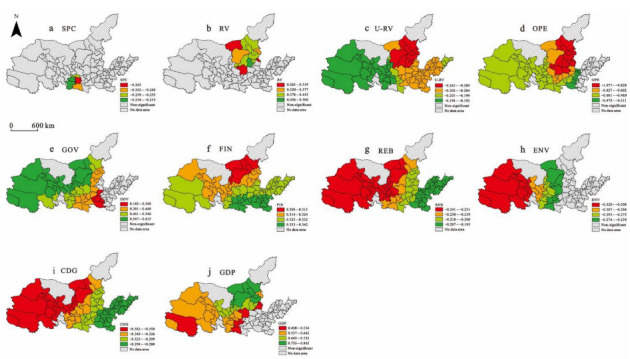
Spatial coefficient distribution of significant variables in 2008–2009.

The regression coefficient of each significant variable is shown in Figure 5. Industrial structure variables had significant effects on resistance, and the characteristics of spatial heterogeneity were obvious. The SPC variable significantly limited regional economic resistance (Figure 5a). The specialized structure had a significant impact on the economic resistance mainly in the central and southern regions of the Yellow River Basin. The value range of this variable was −0.263~−0.235. The negative impact of the financial crisis quickly spread to all industries due to excessive linkages between industries, causing regional economic recession.

The RV variable significantly promoted economic resistance (Figure 5b). RV played a significant role mainly in the central and northern regions of the Yellow River Basin. The improvement in related variety improved the ability of the region to cope with the shock. The value range of the RV was 0.269~0.506. This is mainly because related variety strengthened the connection between different departments. Firstly, related variety dispersed the impact of the shock, avoiding the influence quickly spreading to other industries so as to maintain economic development. Secondly, related diversified industries absorbed the personnel and resource elements of the affected industries, which is conducive to accelerating industrial adjustment and resource reuse.

The U-RV variable had a significant negative effect on regional economic resistance (Figure 5c). The unrelated variety structure in the northeast of the Yellow River Basin had a higher restrictive effect on regional economic resistance. The value range of the U-RV was −0.217~−0.192. The economic development of the Yellow River Basin has long relied on resource-based industries, which were seriously affected by the shock. The proportion of industries unrelated to resources was low. Even though these industries were less affected by the shock, they had less impact on economic development. At the same time, the unrelated variety structure is not conducive to the flow of talents and resources. After the shock, the efficiency of resource utilization was reduced, which is not conducive to economic development.

The OPE variable significantly limited regional economic resistance (Figure 5d). The role of OPE in the east was not significant. The negative effect of OPE was greater in the central area. The value range of the OPE regression coefficient was −1.077~−0.311. This is mainly because the foreign trade of the central and western regions of the Yellow River Basin mainly depends on natural resources. The financial crisis led to a decline in the price and demand of the international resource market, which had an important impact on the resource export industry. This limited the resistance of the central and western regions to the financial crisis.

The GOV variable significantly promoted regional economic resistance (Figure 5e) and showed a spatial heterogeneity characteristic. The regression coefficient increased gradually from the center and south of the Yellow River Basin to the west. The value range of this variable was 0.185~0.615. The results show that in the face of the international financial crisis, government agency helped cities actively resist the shock. There are many resource-based cities in the center and west of the Yellow River Basin, and the state-owned economy accounts for a large proportion. Government agency plays a profound role in the choice of investment and development mode. Government agency can provide funds and policy support for industrial adjustment and help expand the domestic market to digest resource products, thus maintaining economic development and enhancing economic resistance.

The FIN variable had a significant positive effect on regional economic resistance on a global scale (Figure 5f). The regression coefficient increased gradually from the north of the Yellow River Basin to the south. The value range of the FIN regression coefficient was 0.300~0.342. This is mainly because a good financial market can provide enough financial support for industrial adjustment or overcoming difficulties and avoid capital withdrawal after the shock. The government can also attract funds into the market through a series of financial support measures to improve resource utilization efficiency and maintain economic development.

The REB variable significantly limited economic resistance on a global scale (Figure 5g). The regression coefficient decreased gradually from the east of the Yellow River Basin to the west. The value range of the REB regression coefficient was −0.241~−0.195. The financial crisis led to a decline in the price and demand of the international resource market, which had a profound impact on the resource-based industries. There are many resource-based cities in the center and west of the Yellow River Basin, and their economic development depends on resource-based industries, which hindered the ability of the regional economy to resist the shock.

The ENV variable significantly limited regional economic resistance (Figure 5h) and showed a spatial heterogeneity characteristic. The regression coefficient decreased gradually from the center of the Yellow River Basin to the west. The value range of the ENV regression coefficient was −0.320~−0.239. The environmental conditions in these regions play a more significant role in limiting regional resistance to shocks. This is mainly because these regions have long relied on traditional resource industries, which has exacerbated environmental pollution in the process of development. After the financial crisis, the development of traditional resource industries was restricted, and environmental pollution restricted the entry of talents and high-tech industries, resulting in an insufficient foundation for industrial adjustment in these regions, thus limiting the ability to resist the shock.

The CDG variable had a significant negative effect on regional economic resistance on a global scale (Figure 5i). The value range of the CDG regression coefficient was −0.382~−0.208. The urban hierarchy in the west of the Yellow River Basin was more sensitive to economic resistance. This is mainly because high-level cities have more participation in the global division of labor, and the Yellow River Basin is mostly at the supply and processing ends in the global division of labor. After the financial crisis, the international market was depressed, and the high-level cities that participated in the global division of labor were more seriously affected, which hindered the economic resistance.

The GDP had a significant positive effect on regional economic resistance (Figure 5j) and showed a spatial heterogeneity characteristic. The value range of the GDP regression coefficient was 0.420~0.865. Economic development was more sensitive to economic resistance in the north of the Yellow River Basin. A series of supporting measures taken by the region to resist the shock needed the support of local economic development, especially in the center and west of the Yellow River Basin, where economic development is slow.

#### 5.2.2. Recoverability in 2010–2018

**Figure 6:**
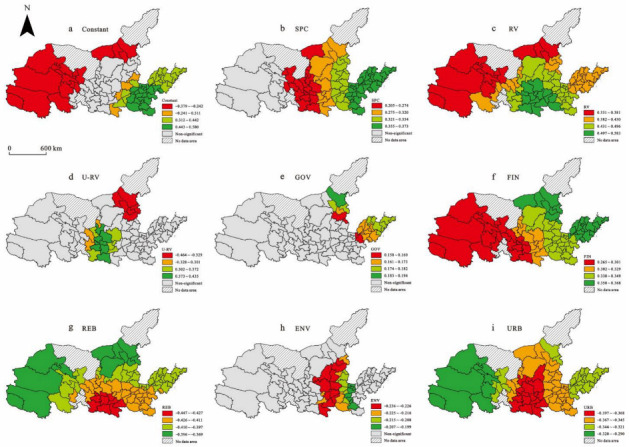
Spatial coefficient distribution of significant variables in 2010–2018.

The regression coefficient of each significant variable is shown in Figure 6. The location reflected by the constant term had a significant effect on the regional economic recoverability on a certain scale (Figure 6a). The location in the west of the Yellow River Basin had a significant negative effect on the recoverability, while the location in the east of the Yellow River Basin had a significant positive effect on the recoverability. The value range of the constant coefficient was −0.379~0.580. This is mainly because the west of the Yellow River Basin has a large proportion of resource industries, the regional environment is poor, and the economic development is relatively slow. This location hindered the adaptation and transformation of the region in the recovery period. However, the east of the Yellow River Basin is a coastal area with good economic development. This location promoted the transformation process of the region in the new environment, and the regional recoverability.

Industrial structure variables had significant effects on recoverability, and the characteristics of spatial heterogeneity were also obvious. The SPC variable significantly promoted regional economic recoverability in the center and east of the Yellow River Basin (Figure 6b); this is contrary to the regression result of resistance. The regression coefficient increased gradually from the center of the Yellow River Basin to the east. The value range of this variable was 0.205~0.373. In the recovery stage, the specialized industrial structure was conducive to the implementation of specific industrial policies, accelerating industrial transformation and improving resource utilization efficiency.

The RV variable significantly promoted economic recoverability on a global scale (Figure 6c). The regression coefficient increased gradually from the northwest of the Yellow River Basin to the southeast. The value range of RV was 0.331~0.583. This is mainly because related variety can promote the generation of innovation activities. When external shocks occur, it can actively adapt to the external environment through inter-industry exchanges, update existing departments, or cultivate new industrial departments.

The U-RV variable had a significant effect on regional economic recoverability on a certain scale (Figure 6d). The unrelated variety structure in the northeast of the Yellow River Basin had a negative effect on recoverability, but it had a positive effect in the center and south of the Yellow River Basin. The value range of U-RV was −0.464~0.435. As China enters the stage of high-quality development, unrelated variety can break the path dependence of regions, achieve path breakthrough, and create new development paths to recover economic development. However, the unrelated variety structure may ignore the role of existing industries, and the cost of cultivating completely unrelated new industries is high, which may have the opposite effect on some regions.

The GOV variable significantly promoted regional economic recoverability in the northeast of the Yellow River Basin (Figure 6e). The value range of this variable was 0.158~0.194. Government agency provided financial and policy support for the breakthrough development of the industry and played an important role in guiding industrial adjustment and accelerating the transformation of the development mode. Especially in the eastern coastal areas, the policy inclination was greater, the economic development was better, and the government’s supporting role was more significant.

The FIN variable had a significant positive effect on regional economic recoverability on a global scale (Figure 6f). The regression coefficient increased gradually from the west of the Yellow River Basin to the east. The value range of the FIN regression coefficient was 0.265~0.368. The good financial market provided enough funds for industrial adjustment and supported the industry in adapting to the new environment.

The REB variable significantly limited economic recoverability on a global scale (Figure 6g). The regression coefficient decreased gradually from the center and south of the Yellow River Basin to the surrounding areas. The value range of the REB regression coefficient was −0.447~−0.369. At this stage, great changes took place in China’s development mode, focusing on the quality of economic development. The past development model based on resource consumption could no longer adapt to the new environment. The large proportion of traditional resource-based industries limited the recovery and development of the regional economy.

The ENV variable significantly limited regional economic recoverability in center of the Yellow River Basin (Figure 6h). The value range of the ENV regression coefficient was −0.236~−0.199. Most of these areas were concentrated in Shaanxi, Shanxi, and Henan. There are many resource-based cities. The past development model seriously damaged the environment. In the recovery stage, this poor environmental condition restricted the investment in industrial technology upgrading, hindered the introduction of high-tech industries, and led to the slow development of the regional transformation.

The URB variable had a significant negative effect on regional economic recoverability on a global scale (Figure 6i). The value range of the URB regression coefficient was −0.397~−0.290. The regression coefficient decreased gradually from the center of the Yellow River Basin to the surrounding areas. This may be related to the measurement of urbanization. We measured urbanization based on the proportion of the non-agricultural population, which cannot fully reflect the quality of urbanization.

## 6. Conclusions

Regional economic resilience is considered to be an important factor to explain and understand the differences in regional performance in coping with and adapting to external shocks [8,26]. Especially under the environment of the COVID-19 epidemic shock, regional economic resilience has attracted an increasing amount of attention from academia and politics [3,17]. In the existing studies, spatial heterogeneity was considered less, and the influence of structural factors on regional economic resilience was mostly analyzed at the macro overall scale. There was less analysis of the elements of agency-based factors. At the same time, the effect of various factors on regional economic resilience has a scale effect; that is, the effect of some influencing factors may only be significant on the local spatial scale, and beyond this scale, it has little effect. Therefore, we took the Yellow River Basin as the research object. We analyzed the spatiotemporal evolution process of economic resilience in the face of the international financial crisis in 2008. On this basis, considering the characteristics of spatial heterogeneity, we analyzed the influence factors of economic resilience in the Yellow River Basin by integrating structural factors, agency-based factors, resource-based economy, and environment and urban hierarchy factors.

Based on our results, we draw the following conclusions:

(1) The resistance of the Yellow River Basin to the financial crisis was high, and it was less affected by the shock in the early stage, but it showed a small decline. The recoverability of the Yellow River Basin after the shock was weak, showing a significant downward trend with the evolution of time. In the long run, the Yellow River Basin could not adapt to the new environment. The economic resilience of the Yellow River Basin showed significant spatial agglomeration and difference characteristics.
(2) In the resistance period and recovery period, the influence mechanism of economic resilience in the Yellow River Basin was significantly different. However, spatial heterogeneity played a significant role in different periods. Related variety, government agency, environment, and opening to the global economy had a significant effect on economic resilience only in a specific small range, beyond which the impact was small. Specialization, unrelated variety, and location had opposite effects in different regions of the Yellow River Basin.
(3) Structural factors still played a significant role in regional economic resilience, but the influence mechanism was changed in different periods. Specialization limited the area’s resistance to shock but enhanced the recoverability after the shock. Related variety significantly improved the regional ability to cope with the shock by giving full play to the “shock absorber”. Unrelated variety was not conducive to regional resistance to the shock and had opposite effects on the recoverability in different regions.
(4) Government agency and financial market significantly promoted the regional economic resistance and recoverability. Environment pollution and resource-based economic structure significantly limited the regional economic resistance and recoverability. Opening to the global economy and urban hierarchy limited the regional resistance to the shock, but strong economic development had the opposite effect of improved regional resistance. The location in the east of the Yellow River Basin enhanced the recoverability; however, the location in the west limited the recoverability.

## 7. Discussion

From the perspective of evolutionary economic geography, considering the dynamic evolution and spatial heterogeneity of basin-scale economic resilience, we discussed the economic resilience mechanism in the face of 2008 international financial crisis by using the MGWR model. For this special scale unit of the Yellow River Basin, we needed to consider the characteristics of spatial heterogeneity and explore the effect of different factors on regional economic resilience at different spatial scales in order to provide a policy reference for the Yellow River Basin to deal with uncertain shocks in the future.

The structural factor is an important factor of regional economic resilience, which has been verified again. In the process of high-quality development of the Yellow River Basin, we need to pay attention to the connection and cooperation between different industries not only to ensure decentralized shocks but also to ensure innovation spillover. Governments should play their role in macro-control and provide policy support for the high-quality development of the Yellow River Basin. Government agency should promote the pace of industrial transformation, accelerate the renewal or creation of regional development paths and promote the transformation process of resource-based cities. The government should enhance the regional specialization of high-tech industries and improve the efficiency of resource utilization. The Yellow River Basin should reasonably control the level of cities, enhance the status of cities in the global division of labor, pay attention to foreign and domestic market, and enhance regional resistance. In view of the unfavorable location conditions such as Qinghai and Gansu, local governments should formulate development strategies considering regional characteristics in future planning. It is worth noting that in the future, we need to implement different development policies on different scales according to the actual urban situation.

Our research also has deficiencies. Due to data limitations, our paper only discussed the economic resilience of the Yellow River Basin in the face of the international financial crisis from the dimensions of resistance and recoverability. At the same time, this paper only selected some key elements to analyze the influence mechanism. In fact, the resilience of the regional economy is affected by many factors. We ignored factors such as innovation and entrepreneurship.

## Figures and Tables

**Table 1:** Description of influencing factors.

Variable	Definition	Unit
Regional economic resilience	Resistance and recoverability index	
Specialization	Specialization index (SPC)	
Related variety	Related variety index (RV)	
Unrelated variety	Unrelated variety index (U-RV)	
Openness	Total import and export/GDP (OPE)	%
Government agency	Fixed asset investment/GDP (GOV)	%
Financial market	Deposits of banking system national/GDP (FIN)	%
Resource-based economy	Proportion of employed persons in mining industry (REB)	%
Environment	Carbon emissions (ENV)	Million tons
Urban hierarchy	0 for urban population less than 0.5 million, 1 for urban population between 0.5 and 1 million, and 2 for urban population greater than 1 million (CDG)	
Urban development	Per capita GDP (GDP)	CNY
Urbanization	Ratio of urban population to total population (URB)	%

**Table 2:** Statistical description of regression coefficient.

	OLS		MGWR	
	Coefficient (2008–2009)	Coefficient (2010–2018)	Bandwidth (2008–2009)	Bandwidth (2010–2018)
Constant	0.000	0.000	85	44
SPC	0.069	−0.075	44	81
RV	0.124	0.252 *	53	79
U-RV	−0.084	−0.113	85	44
OPE	−0.336 **	0.036	57	85
GOV	0.443 ***	0.109	55	83
FIN	0.181	0.312 ***	85	85
REB	−0.122	−0.268 **	85	85
ENV	0.064	−0.086	72	81
CDG	−0.273 **	0.047	85	85
GDP	0.273 *	−0.147	48	85
URB	0.225	−0.244 *	85	85
R^2^	0.418	0.408	0.727	0.696
Log-L	−99.901	−100.624	−66.952	−71.678
AIC	223.802	225.247	191.099	193.483

*** *p* < 0.01; ** *p* < 0.05; * *p* < 0.1.

## Data Availability

Not applicable.
